# Novel Bacterial Metabolite Merochlorin A Demonstrates *in vitro* Activity against Multi-Drug Resistant Methicillin-Resistant *Staphylococcus aureus*

**DOI:** 10.1371/journal.pone.0029439

**Published:** 2012-01-18

**Authors:** George Sakoulas, Sang-Jip Nam, Sandra Loesgen, William Fenical, Paul R. Jensen, Victor Nizet, Mary Hensler

**Affiliations:** 1 Department of Pediatrics, School of Medicine, University of California San Diego, La Jolla, California, United States of America; 2 Center for Marine Biotechnology and Biomedicine, Scripps Institution of Oceanography, University of California San Diego, La Jolla, California, United States of America; 3 School of Pharmacy and Pharmaceutical Sciences, School of Medicine, University of California San Diego, La Jolla, California, United States of America; University of South Dakota, United States of America

## Abstract

**Background:**

We evaluated the *in vitro* activity of a merochlorin A, a novel compound with a unique carbon skeleton, against a spectrum of clinically relevant bacterial pathogens and against previously characterized clinical and laboratory *Staphylococcus aureus* isolates with resistance to numerous antibiotics.

**Methods:**

Merochlorin A was isolated and purified from a marine-derived actinomycete strain CNH189. Susceptibility testing for merochlorin A was performed against previously characterized human pathogens using broth microdilution and agar dilution methods. Cytotoxicity was assayed in tissue culture assays at 24 and 72 hours against human HeLa and mouse sarcoma L929 cell lines.

**Results:**

The structure of as new antibiotic, merochlorin A, was assigned by comprehensive spectroscopic analysis. Merochlorin A demonstrated *in vitro* activity against Gram-positive bacteria, including *Clostridium dificile*, but not against Gram negative bacteria. In *S. aureus*, susceptibility was not affected by ribosomal mutations conferring linezolid resistance, mutations in *dlt* or *mprF* conferring resistance to daptomycin, *accessory gene regulator* knockout mutations, or the development of the vancomycin-intermediate resistant phenotype. Merochlorin A demonstrated rapid bactericidal activity against MRSA. Activity was lost in the presence of 20% serum.

**Conclusions:**

The unique meroterpenoid, merochlorin A demonstrated excellent *in vitro* activity against *S. aureus* and *C. dificile* and did not show cross-resistance to contemporary antibiotics against Gram positive organisms. The activity was, however, markedly reduced in 20% human serum. Future directions for this compound may include evaluation for topical use, coating biomedical devices, or the pursuit of chemically modified derivatives of this compound that retain activity in the presence of serum.

## Introduction

**Figure 1:**
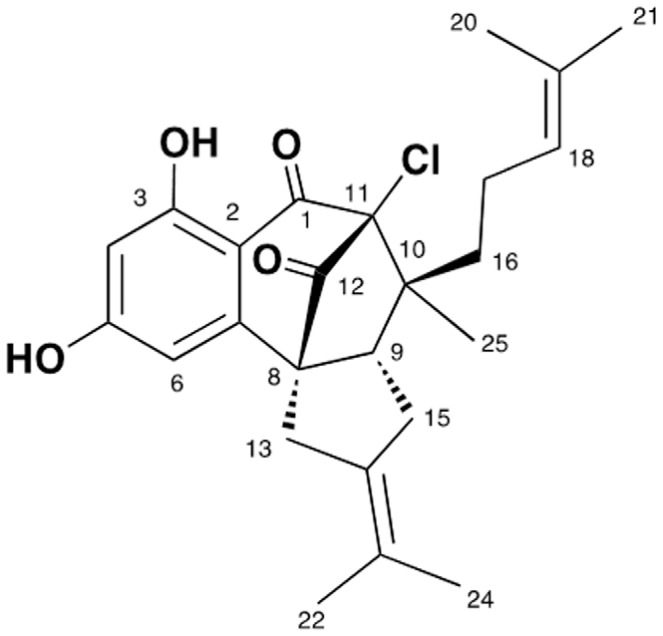
Chemical structure of merochlorin A.

The constant evolution of bacterial pathogens with resistance to clinically available antimicrobial agents drives a continuous demand for the development of novel antimicrobial agents. In the United States, *Staphylococcus aureus* is the leading cause of hospital-associated and community-associated bacterial infections involving the bloodstream, skin and soft tissue and other sites, with a the majority of these pathogens being methicillin-resistant *S. aureus* (MRSA) [1], [2]. MRSA is endemic in most hospitals, and invasive MRSA infections have been associated with higher mortality rates than comparable infections caused by methicillin-susceptible *S. aureus* (MSSA), even when adjusting for host factors, with mortality estimates of about 20% for bacteremia [3]. Currently, the burden of MRSA infections in the United States is enormous, with almost 100,000 annual cases of invasive infections translating to almost 20,000 deaths, representing a leading cause of infection-related mortality by a single pathogen [4]. In addition, *S. aureus* has historically demonstrated a propensity to develop resistance to antibiotics quite rapidly after they are introduced clinically, frequently within 1–2 years [5]. In this setting, the development of novel antimicrobial agents against MRSA continues to be in great demand. We have examined the *in vitro* properties of a novel meroterpenoid, merochlorin A (Figure 1), against MSSA and MRSA with a wide range of resistance determinants against most currently available anti-staphylococcal antibiotics.

## Methods

### Cultivation and extraction

The actinobacterium (strain CNH-189) was isolated from a near-shore marine sediment collected off Oceanside, California. It was identified as a new *Streptomyces* sp. based on 16S rRNA gene sequence analysis (accession number HQ214120). The strain was cultured in sixty 2.8 L Fernbach flasks each containing 1 L of A1 production medium (10 g starch, 4 g yeast extract, 2 g peptone, 1 g CaCO_3_, 40 mg Fe_2_(SO_4_)_3_•4H_2_O, 100 mg KBr) and shaken at 230 rpm at 27°C. After seven days of cultivation, sterilized XAD-16 resin (20 g/L) was added to adsorb the organic products, and the culture and resin were shaken at 215 rpm for 2 hours. The resin was filtered through cheesecloth, washed with deionized water, and eluted with acetone. The acetone was removed under reduced pressure, and the resulting aqueous layer was extracted with EtOAc (3×500 mL). The EtOAc-soluble fraction was dried *in vacuo* to yield 4.5 g of crude extract. Note that no specific permits were required for the collection of ocean floor sediment. The location of sediment collection was not privately-owned or protected territories and there was no impingement on endangered or protected species.

### Purification and Structure Assignment of Merochlorin A

The crude extract was purified by open column chromatography on silica gel (25 g), eluted with a step gradient of dichloromethane and methanol. The dichloromethane/methanol 100∶1 fraction contained a mixture of metabolites, which were purified by reversed-phase HPLC (Phenomenex Luna C-18 (2), 250×100 mm, 2.0 mL/min, 5 mm, 100 Å, UV = 210 nm) using an isocratic solvent system from 85% CH_3_CN to afford merochlorin A. The structure of merochlorin A was assigned by comprehensive spectroscopic analysis involving interpretation of HRMS data (assignment of molecular formula), infrared and UV spectroscopy (functional groups and chromophore analyses), and by comprehensive 1D and 2D NMR studies at 500 MHz.

### Bacterial strains and *in vitro* susceptibility testing

Bacterial strains used in this study are described in Tables 1 and 2 [6]–[15]. Many of these strains have been previously well-characterized in cited references in these tables. Susceptibility testing was performed in duplicate using Mueller-Hinton broth (MHB) and Mueller-Hinton agar (MHA) according to CLSI methods. Susceptibility of merochlorin A and vancomycin against MRSA ATCC33591 was also determined in the presence of 20% activated-pooled human serum (obtained from a pool from healthy donor serum) and 80% MHB. Using 96-well tissue culture treated plates (MICROTEST™ 96, Becton-Dickinson, Franklin Lakes, NJ), MICs in the presence and absence of serum were determined through the broth microdilution method. It was noted that in the presence of serum, the endpoints were difficult to read visually or via a spectrophotometer. Thus, the color-change indicator, resazurin (Sigma-Adrich, St. Louis, MO), was used as an endpoint signal for cell growth as previously shown [16], [17]. Resazurin sodium salt powder was solubilized in sterile water to a final concentration of 270 mg/40 mL, filtered using a Costar #8160 Spin-X® Centrifuge Tube Filter by centrifugation at 10,000 rpm for 10 min, and added to each well of the test plate in a final concentration of 10%. After incubation for 2 hours at 37°C, the plates were visually evaluated for color change from the blue indicator to the pink resorufin, a sign of bacterial growth.

**Table 1:** *In vitro* susceptibility against representative strains of clinically relevant bacterial pathogens for merochlorin A.

	Susceptibility Test MIC (mg/L)	
	Broth Microdilution	Agar Dilution
*Streptococcus pneumoniae* (D39)	2	ND
*Streptococcus pyogenes* (5448, NZ131)	4	ND
*Streptococcus agalactiae* (COH1)	4	ND
*Bacillus cereus*	2	ND
*Staphylococcus epidermidis* (ATCC12228)	4	ND
MRSA (ATCC33591)	2	1
MSSA (ATCC29213)	4	2
Vancomycin-resistant Enterococcus faecium	2	1
Vancomycin-resistant Enterococcus faecalis	1	1
*Clostridium dificile* ATCC 700057	0.3	ND
*Clostridium dificile* (BI)	0.15	ND
*Pseudomonas aeruginosa*	>64	>64
*Escherichia coli* ATCC25922	>64	>64
*Enterobacter cloacae*	>64	>64
*Acinetobacter baumanii*	>64	>64
*Klebsiella pnemoniae*	>64	>64

**Table 2:** *In vitro* susceptibility of merochlorin A against multi-drugresistant *S. aureus* strains, including those resistant to contemporary antibiotics.

Strain Number	Species	Description	merochlorin A MIC (mg/L)
SA7817	MRSA	Progenitor to 7819 [6]	2
SA7819	MRSA	Linezolid resistant, G2756T [6]	2
7819erm	MRSA	*ermC*-transformed 7819 [7]	2
SA354	MRSA	Bloodstream. Progenitor to 355 [8]	2
SA355	MRSA	Linezolid resistant A2500T [8]	2
SA853	MRSA	Progenitor of 853b, VAN MIC 1 mg/L (this study)	2
SA853b	VISA	*In vitro* selected VISA, VAN MIC 8 mg/L (this study)	2
A5937	MRSA	Bloodstream Endocarditis, *mecA*+, VAN MIC 2 mg/L [9]	2
A5940	VISA	Selected *in vivo* VISA, *mecA+*, VAN MIC 4 mg/L [9]	2
SA6300	MRSA	VAN MIC 2 mg/L, progenitor to SA6298 [9]	2
SA6298	VISA	VAN MIC 4 mg/L, selected *in vivo* [9]	2
SA0616	MSSA	Endocarditis, SA701 Progenitor, DAP MIC 0.25 mg/L [10], [11]	2
SA0701	MSSA	DAP MIC 2 mg/L [10], [11]	2
RN6607	MSSA	SA502A, tetM, *agr* group II prototype [12]	4
RN9120	MSSA	*agr* knockout of RN9120 [12]	4
RN9120b	MSSA	VISA selected in vivo from RN9120, VAN MIC 4 mg/L [12]	4
HIP5836	VISA	New Jersey VISA [13]	2
VRSA MI	VRSA	vanA vancomycin-resistant *S. aureus* [14]	2
VRSA PA	VRSA	vanA vancomycin-resistant *S. aureus* [15]	2

Note: Isolates are grouped together above in cases where they have been isolated from the same patient and/or are the results of manipulations in the laboratory of an original strain and have been confirmed identical to each other by pulsed-field gel electrophoresis (PFGE).

### Kill curve assays

Overnight cultures of test strains in MHB were diluted 1∶1000 in fresh broth (inoculum approximately 5×10^5^ cfu/ml) containing merochlorin A at 4× MIC (8–16 mg/L) and incubated with shaking at 200 RPM at 37°C. Samples at time 0, 4, and 24 hours were serially diluted 10^0^–10^6^, and 10 microliters were plated on TSA plates. Colonies were counted after 20 hours at 37°C to calculate surviving cfu/mL.

### Post-antibiotic effect (PAE)

An inoculum of approximately 10^7^ cfu/mL of MRSA ATCC33591 was incubated for 1 hour in MHB containing merochlorin A 2 mg/L (1× MIC) at 37°C with shaking at 200 RPM in a 1.5 mL Eppendorf tube. The bacteria were pelleted by microcentrifugation, washed twice in PBS, and resuspended in an equal volume of fresh antibiotic-free MHB. Samples were obtained at various time points, serially-diluted, and 10 microliters were plated on TSA plates. Colonies were counted at 20 hours to calculate cfu/ml over time. The PAE was calculated according to the Craig and Gudmundsson formula: *PAE = T – C*. In this formula, *T* refers to the time it takes the treated culture to recover by one-log_10_ CFU greater then immediately observed after drug removal (time 0), and *C* refers to the corresponding recovery time observed for the untreated control [18].

### Population analysis

An inoculum of approximately 5×10^7^ cfu/mL of MRSA ATCC33591 was prepared in fresh MHB by transferring several colonies of overnight growth on MHA plates using sterile applicators. Ten microliters of serial 10-fold dilutions (10^0^–10^7^) were plated in triplicate on MHA plates containing 0, 0.25, 0.5, 1, 2, 4, 8, and 16 mg/L of merochlorin A. Colonies were counted at 20 hours, and surviving bacteria were enumerated for each concentration.

### Cytotoxicity assays

Cytotoxcity was assessed by incubation of HeLa and L929 (American Type Culture Collection (ATCC), Manassas, VA) mammalian cell lines (2×10^4^ cells per well) in sterile 96 well tissue culture-treated plates in the presence of decreasing concentrations of merochlorin A and incubation at 37°C with 5% CO_2_ for 24 h. Cytotoxicity was assayed by MTS (3-(4,5-dimethylthiazol-2-yl)-5-(3-carboxymethoxyphenyl)-2-(4-sulfophenyl)-2Htetrazolium) using the CellTiter 96® Aqueous non-radioactive cell proliferation assay according to manufacturer's instructions (Promega Madison, WI). This assay measures the reducing potential of viable cells using a colorimetric reaction, which is quantitated spectrophotometrically at 490 nm. Cytotoxicity was defined as the concentration which showed a >50% reduction in absorbance at 490 nm compared to the merochlorin A-free negative control. Etoposide at 10 mg/L was used as the positive cytotoxicity control.

## Results

### Isolation and structure assignment of merochlorin A

The crude extract (4.5 g) was fractionated by open column chromatography on silica gel (25 g), eluted with a step gradient of dichloromethane and methanol. The dichloromethane/methanol 100∶1 fraction contained a mixture of metabolites, which were purified by reversed-phase HPLC (Phenomenex Luna C-18 (2), 250×100 mm, 2.0 mL/min, 5 mm, 100 Å, UV = 210 nm) using an isocratic solvent system from 85% CH_3_CN to afford merochlorin A (**1**, 12.0 mg), as a pale yellow oil. The molecular formula for merochlorin A was deduced as C_25_H_29_^35^ClO_4_, based on analysis of HRESIMS data (a pseudomolecular ion peak at *m/z* 429.1821 [M+H]^+^) and on interpretation of ^13^C NMR data. Merochlorin A showed strong UV absorptions at 240, 296, and 330 nm, indicating a conjugated aromatic or phenolic functional group. The IR spectrum showed broad absorptions for multiple hydroxyl groups (3380 cm^−1^) and characteristic carbonyl groups (1704 cm^−1^). The ^1^H NMR spectrum of merochlorin A displayed a pair of *meta*-coupled aromatic protons [H-4 (δ_H_ 6.16), H-6 (δ_H_ 6.38)], one olefin proton H-18 (δ_H_ 4.92), five methyl singlets H-21 [(δ_H_ 1.53), H-20 (δ_H_ 1.45), H-24 (δ_H_ 1.65), H-22 (δ_H_ 1.56), H-25 (δ_H_ 0.81)], and an exchangeable proton 3-OH (δ_H_ 11.9). The ^13^C NMR and HSQC spectroscopic data revealed two carbonyl, ten quaternary, four methine, four methyene, and five methyl carbons.

Interpretation of data from 2D NMR experiments allowed three fragments, a 1,2,3,5 tetrasubstituted benzene moiety, the C-10 side chain of the molecule, and a propan-2-ylidenecyclopentane moiety, to be assembled. The first fragment was established from analysis of a ^1^H-^1^H coupling constant and 2D NMR spectral data. The *meta*-coupling of two aromatic protons [H-4 (δ_H_ 6.16, d, *J* = 2.0 Hz), H-6 (δ_H_ 6.38, d, *J* = 2.0 Hz)] indicated a 1,2,3,5 tetrasubstituted benzene moiety. A long range HMBC correlation of the aromatic proton H-4 to carbons C-3 (δ_C_ 165.4) and C-5 (δ_C_ 166.5) and their carbon chemical shifts suggested the substitution of the two hydroxyl groups at C-3 and C-5, respectively. The presence of two hydroxyl groups was confirmed by the methylation of 5-OH followed by the acetylation of 3-OH. A chelated hydroxyl proton (δ_H_ 11.59, 3-OH) strongly suggested the presence of a carbonyl group at C-1, which revealed the ketone group based on the carbon chemical shift of C-1 (d_C_ 193.2). The second fragment, the C-10 side chain of the molecule, was established from COSY crosspeaks and HMBC correlations (Figure 1b). The COSY crosspeaks from the protons H-16 through H-18 [H-16 (δ_H_ 1.40, 1.14)- H-17 (δ_H_ 2.03, 1.75)- H-18 (δ_H_ 4.92)], and the long-range HMBC correlations from an olefinic proton H-18 to carbons C-19 (δ_C_ 131.6), C-20 (δ_C_ 18.1), C-21 (δ_C_ 26.1) permitted the assignment of the side chain of the fragment (C-16 to C-21). The last fragment, a propan-2-ylidenecyclopentane moiety, was assigned by analysis of COSY and HMBC spectroscopic data. The COSY crosspeaks between H-9 and H-15, and long-range HMBC correlations from H-15 to C-8, C-9, C-13, C-14, and C-23: from two dimethyl singlet H-22 to carbons C-14 and C-23: from H-24 to carbons C-14 and C-23 allowed the construction of the propan-2-ylidenecyclopentane moiety.

The three fragments were connected by analysis of HMBC spectroscopic data. The observation of the three bond HMBC correlations from H-6 to C-2, C-4, and C-8 along with two bond HMBC correlations from H-6 to C-5 and C-7 permitted the connection of C-7 to C-8. The connection of C-16 to C-10, of C-10 to C-11, and of C-10 to C-9 was established by the long-range HMBC correlations from H-16 to C-9, C-10, C-11, from H-15 to C-9 and C-10, and from a singlet methyl H-25 to C-9, C-10, C-11, and C-16. The connection of C-12 with C-8 was determined by long-range HMBC correlations from H-13 to C-8 and C-12 and from H-9 to C-8 and C-12. Establishing the connectivity of the remaining part of the molecule was difficult due to the absence of any protons correlating with carbons C-1, C-11, and C-12. Thus, we considered carefully the chemical shifts of C-1 (δ_C_ 193.2), C-12 (δ_C_ 200.1) and C-11 (δ_C_ 91.1): these data led us to place the remaining chlorine atom at C-11 and to connect C-1 to C-11 and C-11 to C-12, thus completing the structure assignment of merochlorin A.

*In vitro* susceptibility testing using both broth and agar-based CLSI methods against single strain representatives of clinically-relevant Gram-positive and Gram-negative bacteria revealed activity of compound merochlorin A against MSSA, MRSA, and VRE but no activity against Gram-negatives (Table 1). For bacteria for which activity was present, agar dilution consistently gave a single dilution lower MIC than broth microdilution (Table 1). Of particular interest was the high potency against *Clostridium dificile* representative strains, including the contemporary virulent BI strain (also known as the NAP1 or ribotype 027 strain).

**Figure 2:**
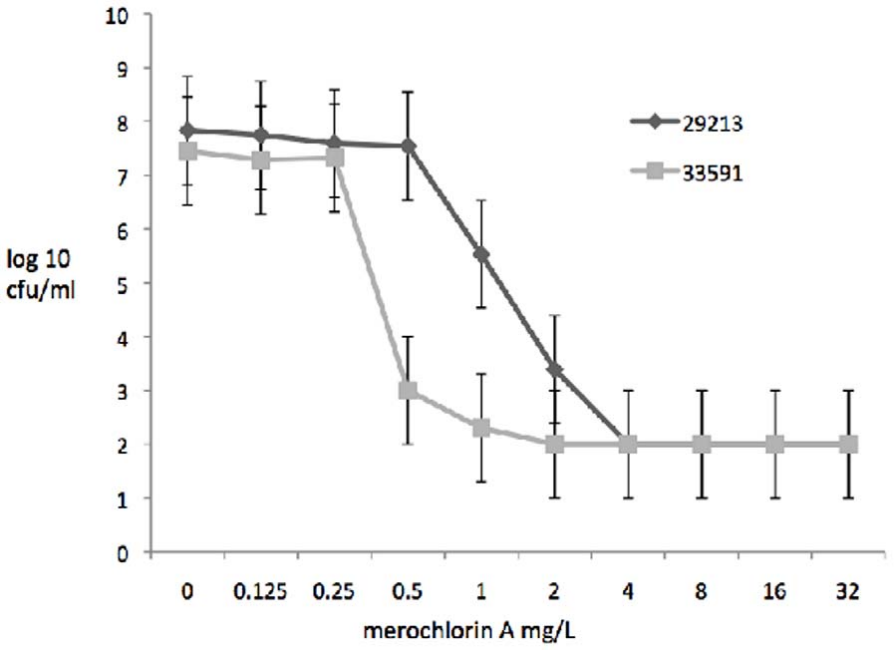
Merochlorin A population analysis of ATCC 29213 (MSSA) and ATCC 33591 (MRSA). Significantly higher viable bacteria at 0.5 and 1 mg/L for MSSA compared to MRSA (p<0.01).

Susceptibility testing using various MRSA and MSSA with resistance to contemporary antibiotics revealed no significant change in MIC to merochlorin A with the acquisition of resistance to vancomycin (both intermediate-level VISA and high–level vanA-mediated VRSA), linezolid, or daptomycin (Table 2). The MIC of all *S. aureus* strains tested against merochlorin A was 2–4 mg/L. Population analysis of ATCC 29213 (MSSA) and ATCC 33591 (MRSA) showed a slightly heterogeneous type of susceptibility for the MSSA and a homogeneous susceptibility for MRSA (Figure 2).

Examination of an accessory gene regulator (*agr*) knockout of MSSA RN9120, derived from strain RN6390b (SA502A) (Table 2) which has previously demonstrated a proclivity towards development of vancomycin intermediate resistant phenotype also did not show a change in merochlorin A MIC. Agar dilution susceptibility testing showed one dilution lower MIC than broth-based methods (data not shown).

Cytotoxicity, defined as a 50% reduction in absorbance at 490 nm measuring the reduced MTS reagent, was noted at 64 mg/L and 2 mg/L in HeLa cells at 24 and 72 hours, respectively, and at 32 mg/L and 4 mg/L in L929 cells at 24 and 72 hours, respectively.

However, merochlorion A susceptibility testing in MHB containing 10% and 20% human serum resulted in complete inactivation of *in vitro* activity (MIC>64 mg/L) for all strains tested. The post antibiotic effect against MRSA ATCC33591 was determined to be 8 and 9 hours at 2 mg/L (1× MIC) in 2 separate experiments.

Killing assays were performed in Mueller-Hinton broth against linezolid-resistant MRSA and 2 VISA (one mecA+ and one mecA−). For the VISA strains, fully vancomycin-susceptible progenitor parent strains were compared in parallel. For all strains tested, merochlorin A exhibited potent bactericidal activity, achieving the limit of detection at 24 hours (approximately 10^4^ cfu/mL reduction). For both pairs tested, the VISA isolate showed increased killing at 4 hours compared to the vancomycin susceptible parent strain (data not shown).

## Discussion

We report a novel natural product, merochlorin A, with *in vitro* activity against multi-drug resistant Gram-positive organisms, including *C. dificile* and MRSA. Merochlorin A (Figure 1) possesses a new carbon skeleton unrelated to any antibacterial agents reported to date. This is particularly noteworthy given the paucity of novel antibiotic classes currently in development for clinical use. Resistance to commonly-used MRSA agents, including vancomycin (both intermediate and high-level vanA-mediated), linezolid, and daptomycin did not affect *in vitro* susceptibility. We evaluated resistant isolates to contemporary anti-staphylococcal antibiotics because future agents must be able to counter resistance to these drugs, which is to be expected in the years ahead with increased utilization. While resistance to linezolid and daptomycin is rare among clinical isolates, we anticipate higher resistance rates in the future. Furthermore, reduced susceptibility across classes that has been determined between the lipopeptide daptomycin and intermediate-level glycopeptides resistance in *S. aureus*, as well as reduced susceptibility to telavancin mediated by vanA, further heighten concern about the future of antimicrobial resistance in *S. aureus*. This lack of cross-resistance of merochlorin A to contemporary antibiotics with specific mechanisms of action is consistent with our preliminary mechanism of action studies suggesting global inhibition of DNA, RNA, protein, and cell wall synthesis.

Merochlorin A showed bactericidal activity and had an 8–9 hour post antibiotic effect. Cytotoxicity was favorable in the 24 hour assay but was increased to concentrations close to its antimicrobial range in the 72 hour assay. Population analyses of a representative MRSA and MSSA showed a more heterogeneous susceptibility profile for MSSA and a homogeneous profile for MRSA.

In light of inhibition by serum, several avenues exist for merochlorin A. First would be chemical modification of the compound in order to eliminate serum inactivation while preserving *in vitro* activity against MRSA. Our laboratories have started to develop derivatives based on merochlorin A structure that are not inhibited by serum and achieve compounds better suited for systemic use. Other strategies may include development and formulation as an enterally administered agent against *C. dificile*, topical *S. aureus* decolonization, or the topical therapy of wound infections, particularly in the setting of rising resistance to mupirocin in *S. aureus* [19]. Note that prolonged treatment of infected devitalized wounds with systemic vancomycin was a feature of some cases in which patients became colonized with vancomycin-resistant *S. aureus* [14], [15]. Considerations can also be made for using this compound to coat biomedical devices such as central venous catheters that are at risk for becoming colonized and subsequently infected with resistant Gram-positive pathogens such as MRSA and *S. epidermidis*.

In summary, we present merochlorinA, a novel meroterpenoid compound which may provide a foundation for developing viable antimicrobial compounds against MRSA and *Clostridium dificile* that are unaffected by resistance to currently utilized antimicrobial classes.
